# Genotyping by sequencing reveals a chromosome A04 QTL governing whitefly resistance in upland cotton

**DOI:** 10.1038/s41598-025-32937-4

**Published:** 2025-12-17

**Authors:** Obad Ul Rahman, Syed Bilal Hussain, Muhammad Javed, Sarmad Frogh Arshad, Zulqurnain Khan, Muhammad Usman, Hasan Junaid Arshad, Muhammad Anwar

**Affiliations:** 1 Institute of Molecular Biology and Biotechnology (IMBB), Bahauddin Zakariya University Multan, Multan, 60000 Pakistan; 2 Patron Seeds, Multan, 60000 Pakistan; 3 MNS University of Agriculture, Old Shujaabad road, Multan, Pakistan; 4 Hainan University, Haikou, China

**Keywords:** GBS, SNP, Whitefly resistance, At4g27190, RPPL1, Biotechnology, Molecular biology, Plant sciences

## Abstract

Cotton plants utilize physical defenses such as trichomes and leaf characteristics to deter insect pests and environmental stressors, safeguarding their growth and productivity. Cotton insect pests such as whiteflies, aphids, jassids and boll weevils not only directly damage the crop but also transmit harmful viruses, leading to substantial yield and quality loss. A GBS study was conducted on 206 cotton plants from a cross between whitefly resistant and whitefly susceptible varieties to identify genetic markers linked to whitefly resistance and yield traits. This study identified genetic regions associated with whitefly resistance and yield traits, including specific genes on chromosomes A04 and D09 linked to resistance mechanisms and flower number, respectively. This study identified genes involved in whitefly resistance (*At4g27190* and *RPPL1*) and genes linked to increased flowering and plant vigor (GUS1, MBD4L), paving the way for the development of molecular marker-assisted breeding to create resilient cotton cultivars.

**Supplementary Information:**

The online version contains supplementary material available at 10.1038/s41598-025-32937-4.

## Introduction

GBS is a powerful technique for efficiently identifying SNPs across large numbers of samples, enabling genome-wide or tagged genetic analysis^1,2^. Restriction enzymes are used to cut DNA into specific fragments, which are then tagged with unique barcodes to identify their origin after being pooled for sequencing^3,4^. Next-generation sequencing was used to sequence the DNA fragments, and the barcode information was used to identify and analyze specific SNP variations within the sequenced data^5,6^. GBS is a cost-effective method for identifying genes linked to disease resistance, yield, and complex traits, as well as for studying genetic variation within and between populations and assessing gene expression^5^. GBS employs restriction enzymes to fragment DNA, targeting specific regions while avoiding repetitive sequences, and attaches unique barcodes to the fragments for identification after pooling^7,8^. GBS involves fragmenting DNA with restriction enzymes, adding barcodes, performing PCR amplification, sequencing, and analysis to identify SNPs, making GBS a valuable tool for plant and animal breeding, genetic studies, and genome-wide association studies^9,10^. The choice of restriction enzymes (ApeKI, PstI, and EcoRI) in cotton GBS studies was influenced by their ability to target specific DNA regions and avoid repetitive sequences, with EcoRI being less commonly used owing to its less frequent cutting^11,12^. The selection of restriction enzymes in GBS, such as ApeKI with its recognition sequence GCWTC, is based on factors such as genome coverage, fragment size, and GC content to optimize the analysis in cotton genotyping studies^2,13,14^. ApeKI is commonly used in GBS because of its frequent cutting, suitable fragment size, and low cost, resulting in good genome coverage and informative marker distribution^15^. GBS employs adaptors with specific sequences for different sequencing platforms, including RE overhang, spacer and sequencing primer sites, to facilitate DNA fragment ligation and sequencing initiation, while barcodes are used to identify and differentiate between samples during the workflow^16–20^. Barcodes are unique nucleotide sequences assigned to samples to prevent misidentification during GBS, whereas SNPs are single-nucleotide variations within a genome, such as a change from C to G in a DNA sequence, occurring at a frequency of approximately one per 1000 nucleotides^1,21–23^. Millions of SNPs exist in complex organisms such as humans, with some influencing disease susceptibility and traits, such as plant response to pesticides or disease resistance. Identifying these SNPs in polyploid cotton can help link them to diseases and yield traits, enabling the selection of resistant genotypes with specific SNP alleles^24–26^. SNPs offer more detailed genetic resolution than markers such as SSRs do, enabling the precise identification of genes linked to traits such as disease resistance and yield. High-throughput sequencing has made SNP analysis cost-effective, facilitating the discovery of whitefly resistance-related SNPs and the identification of nearby candidate genes involved in defense mechanisms^27–29^. Identifying whitefly resistance-related SNPs through GBS enables marker-assisted selection of resistant genotypes, reduces field exposure and pesticide dependency, and paves the way for developing genetically resilient cotton varieties^30^. QTLs are specific chromosomal regions containing multiple genes that contribute to traits such as whitefly resistance and are identified through statistical analysis of genetic markers and phenotypic data from crosses between resistant and susceptible parents, revealing the complex genetic architecture of resistance^31–33^. QTL mapping identifies chromosomal regions containing multiple genes involved in complex traits such as whitefly resistance, aiding in the development of marker-assisted selection programs for breeding resistant cotton varieties by utilizing DNA markers linked to these QTLs^34,35^. Integrating SNP-based association mapping, GBS, and QTL mapping provides a comprehensive understanding of whitefly resistance genetics in cotton, with QTL mapping being crucial for developing resistant varieties because of the absence of a complete SNP-mapped QTL reference map^36^. GBS can identify whitefly resistance genes by comparing resistant and susceptible genotypes, and SNP markers can help elucidate the function of genes such as Mi-1.2 in cotton defense. Studying gene expression in whitefly infested cotton can further reveal resistance mechanisms^37–39^. Comparing upregulated genes in resistant and susceptible cotton genotypes during whitefly infestation, along with identifying resistance markers using GBS, can lead to the development of new, highly resistant cotton varieties with reduced pesticides dependency by validating candidate genes and discovering additional resistance genes and pathways.^30,40^. By continuing research on whitefly resistance in cotton, scientists have aimed to develop long-lasting, whitefly immune varieties and accessible DNA markers for large-scale breeding programs, leading to a more sustainable cotton production system^41^.

## Materials and methods

First, during the cropping season of 2021–22, we screened both resistant and susceptible parents for whitefly resistance^42^. Two resistant parents, namely, CA-12 and AGC-555, and two susceptible parents, namely, GOMAL-105 and SLS-87/175, were screened for whitefly resistance and susceptibility. In the next cropping season, 2022–23, these selected parents were crossed such that CA-12 was crossed with GOMAL-105, and AGC-555 was crossed with SLS-87/175^43^. After that, the F1 generation was grown in a glasshouse to obtain seeds to grow F2 in the next cropping season^44^. In the next cropping season, 2023–24, the F2 generation was grown in the field and evaluated for phenotypic data and genotypic data. The F2 generation obtained from a cross between CA-12 and GOMAL-105 was subjected to further studies, and the F2 generation obtained from a cross between AGC-555 and SLS-87/175 was discarded because more than 50% sterile plants were present^45,46^. The F2 germplasm was cultivated during the 2023–24 cropping season on 200 m^2^ at the research farms of Patron Seeds (71°21’30” E, 29°59’30” N). Cotton beds measuring 60 cm in length with 30 cm furrows were prepared via a bed planter. Seeds were manually sown (Chopa method) in a zigzag pattern along both edges of the beds at a spacing of 30 cm per plant^47^. Since non-delinted cottonseeds were used, 3–4 seeds were sown per chopa to increase germination rates^48^. Following sowing, standard agronomic practices were implemented to ensure optimal crop growth, except for pesticide application for whitefly control^49^. Young leaves from 30-day-old plants were collected, frozen, and stored for DNA extraction via the CTAB method to identify genetic variations^50^. Phenotypic data was recorded as follows: Days to First Flower (DTF) data was collected daily after 35 days of sowing and continued until 47 days post-sowing^51^. Data regarding Flowers/plants (FP) was collected daily over a period of three weeks, commencing 35 to 47 days following sowing, for all genotypes^52^. We collected morphological data for Nodes to 1st monopodia (NTM), Monopodia/Plant (MP), Sympodia/plant (SP), Leaf Length (LL), Leaf Width (LW), and Petiole Length (PL) after 90 days of sowing. Additionally, we calculated Leaf Area (LA) using the Grid Method of Leaf Area Measurement^53^. The tolerance data for Whitefly adults and nymphs, commonly known as the Whitefly count (WC), were taken from the field 60 days after sowing. The data was obtained three times, each with a 30-day interval. Whitefly data was acquired by randomly selecting three plants and counting whitefly adults and nymphs on the upper leaf of the first plant, the middle leaf of the second plant, and the lower leaf of the third plant^54^. The data for plant height (PH) and yield parameters were recorded at maturity, including Bolls/plant (BP), Bolls weight (BW), Yield/plant (YP)^55^. Fibre attributes were calculated following crop picking, including seed index (SI), lint index (LI), ginning outturn (GOT), and staple length (SL)^56^. DNA purity was assessed via spectrophotometry and gel electrophoresis, and the DNA samples were subsequently sent for GBS analysis following the protocol of Elshire^57^ (USA)^58,59^. Library preparation for GBS was carried out by using high-quality DNA extracted from plant tissues, and the ApeK1 enzyme with the GCWTC recognition site was used to cut DNA^14,50,57,60^. Frequent cutting, suitable fragment size and low cost were the causes of ApeKI selection in GBS. Adapters having primer sites and barcodes were ligated with DNA fragments for sample identification and PCR amplification^15^ Sequencing was performed on Illumina platforms using 50–150 bp reads, with sequencing-by-synthesis chemistry detecting fluorescently labeled nucleotides to generate raw FASTQ data^61,62^. After sequencing, barcodes were used to identify sample origins and reads aligned to a reference genome to identify SNPs and other genetic variations^6,63–65^. Bioinformatics tools were used to identify and confirm SNPs across the samples, enabling the analysis of genetic diversity, population structure, and trait associations within the cotton germplasm^66^. Raw sequence data from GBS, consisting of millions of short DNA fragments (50–150 bp) in Fastq format, were processed via bioinformatics tools to identify barcodes and differentiate reads from different pooled cotton samples^67,68^. Fragment size distribution was measured, and GBS data were aligned to a reference cotton genome to identify SNPs and other genetic variations, enabling the analysis of genetic diversity, population structure, and trait associations within the cotton germplasm^69–71^. Comparing identified SNPs with phenotypic data helps identify those associated with yield-related traits and whitefly resistance. Aligning SNPs to the genome determines their exact location and predicts their functions, whereas variation in alignment across samples reveals individual differences. Specialized software is used for accurate GBS data alignment because of the complexity of the cotton genome^72–74^. Burrows wheeler aligner (BWA) was used to align GBS data, followed by quality control using variant calling tools to identify and filter low-quality SNPs, which were then annotated to understand their potential functions^75,76^. Annotation tools associate SNPs with specific genomic regions, including coding, regulatory, and noncoding regions, to predict their potential impact on gene expression, protein function, and cellular processes, aiding in the identification of disease-related SNPs and understanding their functional significance^77–79^. Annotation in GBS adds biological context to SNPs, pinpointing their exact location and linking them to specific genes to understand their potential impact on phenotypes and diseases, aiding in prioritizing SNPs for further analysis and improving reference genomes and annotation databases^80–83^. Bioinformatics tools such as TASSEL-GBS, TASSEL, JOINMAP, Win QTL Cartographer, and MAPCHART were used for preprocessing, quality control, demultiplexing, alignment, variant calling (SNP detection), and downstream analysis, including QTL detection^84–86^. TASSEL is a bioinformatics tool used for analyzing GBS data, including SNP calling, database building, barcode processing, read alignment, SNP discovery, quality filtering, population structure analysis, GWAS, and MAS, and requires computational resources for large datasets^87–89^. TASSEL-GBS, a specialized tool for plant breeding, uses SNP calling algorithms to identify SNPs from aligned reads in its database, with alignment to a reference genome being a crucial step in the SNP discovery process^90,91^. TASSEL-GBS uses its own internal alignment engine for SNP calling, unlike other pipelines, which offer choices such as BWA, which is a tool for aligning reads to a reference genome^92^. SNP filtering was performed via TASSEL on the basis of minor allele frequency, missing data rate, coverage depth, segregation distortion, and heterozygosity to improve data quality and reduce the computational burden. The remaining 8,889 SNPs were imputed via LD-kNNi in TASSEL, with imputation accuracy assessed by masking known genotypes and comparing them to predicted alleles^93^. Highly filtered SNP data in VCF format were used to construct a linkage map by calculating recombination frequencies between linked markers via algorithms such as the Kruskal‒Wallis test and regression, which were used to assess their separation during meiosis^94–96^. JOINMAP was used to construct a linkage map via high-quality SNP data and pedigree information; this map represents the linear order of markers within linkage groups on the basis of recombination frequencies; facilitates assessments of map length, marker distribution, and potential genotyping errors; and serves as a foundation for QTL studies and marker-assisted selection^97–99^. A high-density linkage map was constructed using recombination frequencies (Kruskal‒Wallis test) and visualized (MapChart), enabling marker-assisted selection.

WinQTL Cartographer2.5_011 (https://brcwebportal.cos.ncsu.edu/qtlcart/WQTLCart.htm) was used to perform composite interval mapping (CIM) analysis on a genetic linkage map (constructed from SNP information) to identify QTLs associated with the traits of interest, utilizing phenotypic data^100,101^. QTLs were designated and prioritized for further analysis on the basis of their highest LOD scores and phenotypic variances (R2) and were named via a convention (e.g., qPH for plant height, qFP for flowers per plant, and qWC for whitefly count) after being identified via a 1000-iteration permutation procedure to determine the LOD threshold for each trait^38,102^. QTLs were designated via a naming convention (e.g., qPH for plant height, qFP for flower per plant, and qWC for whitefly count) and prioritized for further analysis on the basis of their highest logarithm of odds (LOD) scores and phenotypic variations (R2). Genes within identified quantitative trait loci (QTLs) were positioned on the basis of genetic distances on the map, followed by gene mining with Cotton FGD (https://cottonfgd.org/) to identify 377 candidate genes in detail in Supplementary Tables 1 & 2. Prioritization of these candidates for whitefly resistance was achieved via TAIR (https://www.arabidopsis.org/), and relevant literature was used to assess their predicted functional roles^103^. The Mi 1.2 genes in *Solanum lycopersicum*, which were identified as key factors in tomato whitefly resistance, suggested that their cotton orthologs may also play a role in whitefly resistance^104,105^. The mRNA sequence of the Mi 1.2 gene (NM_001247134.1) was used in a BlastX search against the TAIR 10 protein database to identify homologous genes in *A. thaliana*, followed by a similar BlastX search of 211 *G. hirsutum* genes to identify homologs of known whitefly resistance genes.

## Results

### Genetic linkage map

A total of 3375 SNPs were identified in the F2 population via GBS, with 1793 SNPs selected on the basis of missing data and heterozygosity. After 996 SNPs were converted to the ABH genotype, a linkage map was constructed via JOINPAM 4.0^106^. The groups were established with a minimum logarithm of odds (LOD) threshold of 10.0 marker orders and were estimated via the regression mapping algorithm. The recombination fractions were converted to map distances via the Kosambi mapping function^107^. The strongest cross-link (SCL) information in combination with the known mapped locations of the ungrouped SNPs was used to merge these markers with the clearly identified linkage groups of *G. hirsutum.*^108^.

These 996 SNPs were analyzed via JOINMAP 4.0 software. The linkage group output and map position were subsequently determined through MapChart 2.2 for Windows, and the process was used to compute and display 3-D map-based marker distances^109,110^. One hundred thirty (130) SNPs were distributed in 19 linkage groups. The remaining 866 SNPs were not linked and were excluded from the maps or linkage groups. The LGs were numbered (1–19) on the basis of the assigned chromosome numbers^111^.

**Fig. 1:**
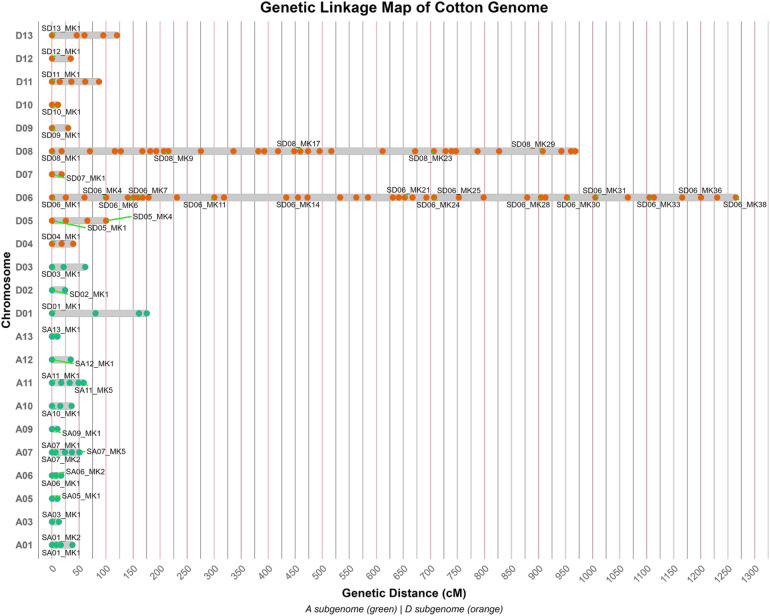
Genetic linkage map of tetraploid cotton. (**A**) Chromosomes are ordered vertically with the D subgenome (orange, left) and A subgenome (green, right). (**B**) Scale bar indicates genetic distance in cM. (**C**) Filled circles represent molecular markers with positions determined by recombination frequency. (**D**) Absence of A02, A04, and A08 chromosomes reflects insufficient marker coverage for these linkage groups.

The LODs of the markers/loci ranged from 2 to 10. The basic information of the linkage groups (LGs) is presented in (Table 1). The current map spanned only 3213.3 cM, with an average marker density of 2.28 cM^112^. The genetic length of the LGs ranged from 10.1 cM (Chr/LG A3, Chr/LG A13) to 1265 cM (Chr/LG D06). On average, one linkage group presented approximately 38 SNP markers that covered an average of 34.2 cM. The most common marker-covered linkage group was 38 (Chr/LGD06), which had 38 markers with an average marker density of 0.03 cM. In contrast, linkage groups A09, A12, A13, D02, D07, D09, D10 and D12 each had the lowest number of SNP markers (only 2; Table 1; Fig. 1).

**Table 1:** Summary statistics of the *genetic linkage map of G. hirsutum*. This table provides an over view of the genetic linkage map constructed for *G. hirsutum*. Each row represents a specific linkage group (chromosome), and the columns provide the following information: group: the chromosome identifier. Marker_num: the number of markers mapped to the chromosome. Map_Len(cM): the total genetic length of the chromosome in centimorgans (cM). Marker_Density (markers/cM) Ave_Interval (cM): the average distance between adjacent markers on the chromosome. Max_Gap(cM): the maximum genetic distance between any two adjacent markers on the chromosome.

Subgenome	Chromosome	Marker count	Map length (cM)	Marker density (markers/cM)	Average interval (cM)	Maximum gap (cM)
A	A01	4	37.8	0.11	12.6	21.5
A	A03	2	12.8	0.16	12.8	12.8
A	A05	2	10.1	0.2	10.1	10.1
A	A06	3	17.2	0.17	8.6	9.8
A	A07	5	50.8	0.1	12.7	17.4
A	A09	2	10.1	0.2	10.1	10.1
A	A10	3	36.3	0.08	18.1	21
A	A11	5	58.7	0.09	14.7	17
A	A12	2	34.7	0.06	34.7	34.7
A	A13	2	10.1	0.2	10.1	10.1
D	D01	4	175.3	0.02	58.4	80.5
D	D02	2	24.2	0.08	24.2	24.2
D	D03	3	61.5	0.05	30.7	39.9
D	D04	3	39.4	0.08	19.7	21.5
D	D05	4	100.1	0.04	33.4	39.9
D	D06	38	1265	0.03	34.2	115.1
D	D07	2	17.8	0.11	17.8	17.8
D	D08	32	968.2	0.03	31.2	94.9
D	D09	2	29.9	0.07	29.9	29.9
D	D10	2	11.2	0.18	11.2	11.2
D	D11	5	87	0.06	21.8	25.5
D	D12	2	34.7	0.06	34.7	34.7
D	D13	5	120.4	0.04	30.1	45.8
Total		134	3213.3	2.22	22.69	115.1

### QTL analysis

**Fig. 2:**
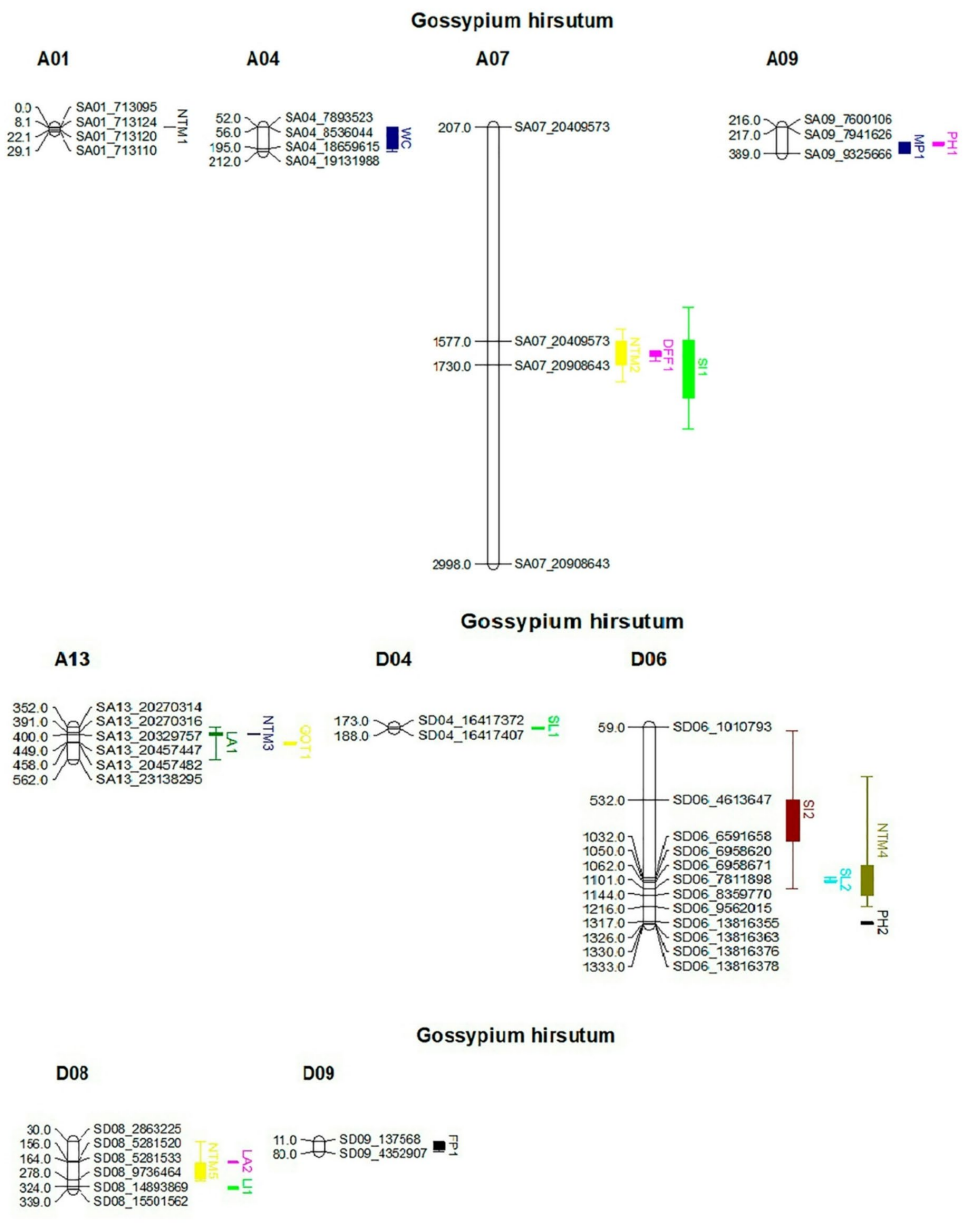
This figure presents a genetic map of cotton *(Gossypium hirsutum)* for chromosomes A01, A04, A07, A09, A13, D04, D06, D08 and D09. The map was constructed via MapChart. The following markers associated with specific traits are highlighted: chromosome A01: black marker: nodes to 1st monopodia 1 (NTM1) chromosome A04: purple marker: whitefly count (WC) chromosome A07: yellow marker: nodes to 1st monopodia 2 (NTM2); pink marker: days to 1st flowering (DFF); green marker: seed index (SI) chromosome A09: blue marker: monopodia per plant 1 (MP1) pink marker: plant height 1 (PH1) chromosome A13: white marker: leaf area 1 (LA1) blue marker: nodes to 1st monopodia 3 (NTM3) yellow marker: beginning 1 (GOT1) chromosome D04: light green marker: staple length 1 (SL1); chromosome D06: Burgundy marker: seed index 2 (SI2) cyan marker: staple length 2 (SL2) olive marker: nodes to 1st monopodia 4 (NTM4) chromosome D08: yellow marker: nodes to 1st monopodia 5 (NTM5) pink marker: leafy area 2 (LA2) olive marker.

**Fig. 3:**
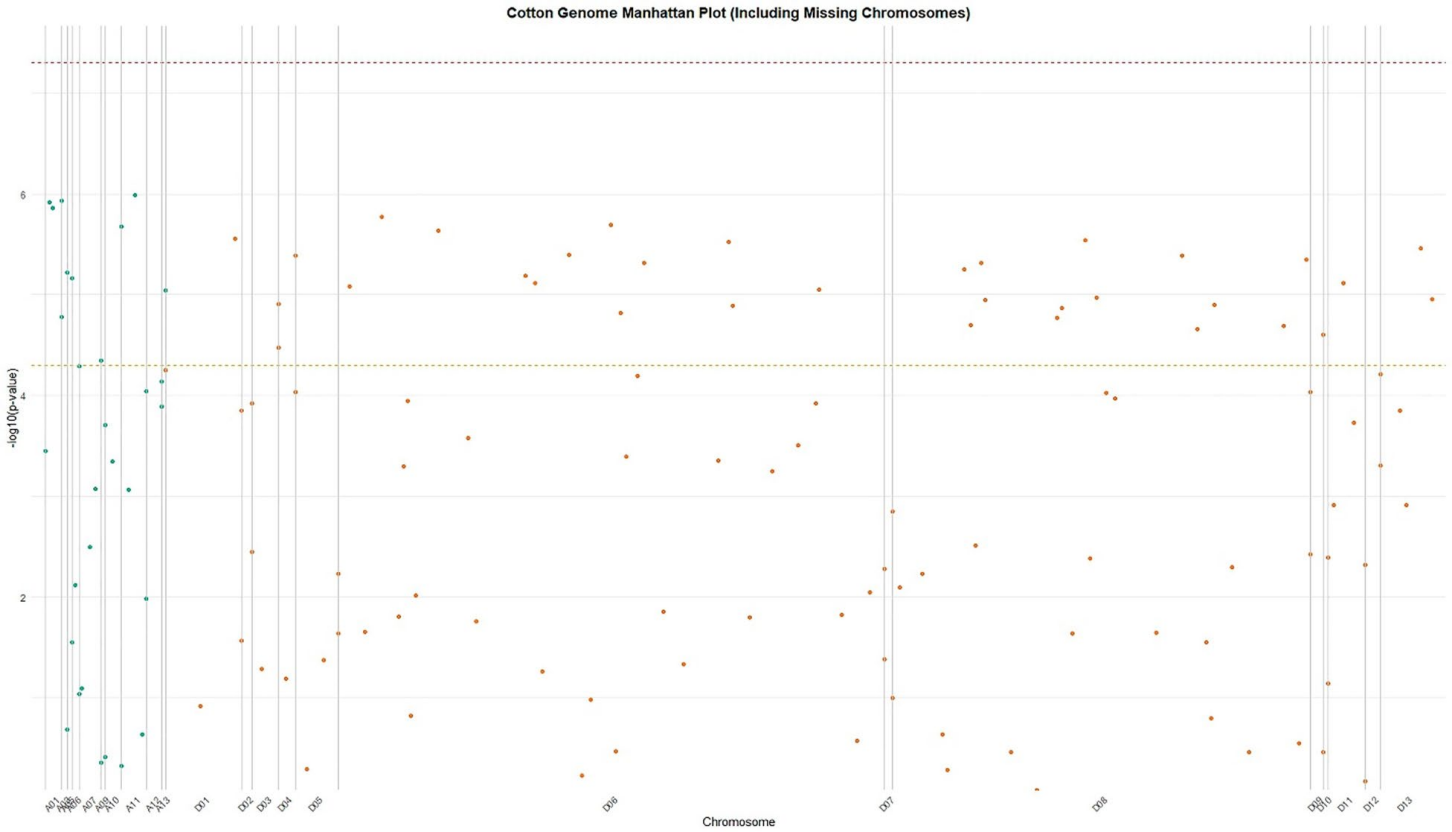
Genome-wide association study (GWAS) Manhattan plot of Cotton genome variants. The plot displays -log10 (p-values) of genetic associations across chromosomes (x-axis) with phenotypic traits of interest. Chromosomes are alternately colored for clarity (odd-numbered in blue, even-numbered in orange). The horizontal red line indicates the genome-wide significance threshold (*p* < 5 × 10^-8). Several peaks above the threshold suggest potential candidate loci for further investigation. Note: Some chromosomal positions show missing data points due to technical limitations in variant calling.

Five QTLs for Nodes to 1st Monopodia were identified on chromosomes 1, 7, 13, 19 and 21, designated as qNTM1_A01, qNTM2_A07, qNTM3_A13, qNTM4_D06, and qNTM5_D08, respectively^113–116^ (Table 2). QTLs for whitefly count (qWC1_A04), flower per plant (qFP1_D09), nodes to 1st monopodia (qNTM5_D08), and plant height (qPH1_A09) were identified on chromosomes A04, D09, D08 and A10, respectively, with LOD scores of 4.980, 5.944, 11.560 and 6.202 and phenotypic variances of 0.250, 0.298, 0.263 and 0.211^117,118^ (Fig. 2). The QTLs qWC1_A04 and qFP1_D09 were further investigated to identify genes associated with whitefly resistance and flowers per plant, respectively (Fig. 3).

**Table 2:** Quantitative trait loci (QTLs) associated with yield and fiber quality traits in *Gossypium hirsutum*. This table presents a comprehensive list of QTLs identified for yield and fiber quality traits in *G. hirsutum*. Each QTL is characterized by the following information: chr: chromosome number position (cM): the genetic position of the QTL in centimorgans start bp; the starting base pair position of the QTL end bp; the ending base pair position of the QTL No. of genes; the number of genes within the QTL interval LOD; the logarithm of the odds ratio, indicating the strength of the QTL effect R2; and the proportion of phenotypic variance explained by the QTL Additive_effect: the additive effect of the QTL allele Dominant_effect: the dominant effect of the QTL allele. qNTM1_A01 nodes to 1st monopodia 1, qWC1_A04 whitefly count, qNTM2_A07 nodes to 1st monopodia 2, qDFF 1_A07 days to 1st flowering, qSI1_A07 seed index, qPH1_A09 plant height 1, qMP1_A09 monopodia per plant 1, qWC2_A09 whitefly count 2, qNTM3_A13 nodes to 1st monopodia 3, qLA1_A13 leaf area 1, qGOT1_A13 ginning outturn 1, qSL1_D04Staple length 1, qPH2_D06 plant height 2, qNTM4_D06 nodes to 1st monopodia 4, qSL2_D06 staple length 2, qSI2_D06 seed index 2, qNTM5_D08 nodes to 1st monopodia 5, qLA2_D08 leaf area 2, qLI1_D08 Lint index 1, qFP1_D09 flowers per plant 1.

Comprehensive QTLs information									
QTLs	Chr	Position(cM)	Start bp	End bp	No of genes	LOD	R2	Additive_effect	Dominant_effect
qNTM1_A01	1	101.01	3,740,119	4,720,876	65	15.048	0.262	2.647	2.928
qWC1_A04	4	211.01	7,893,523	19,131,988	211	4.980	0.250	15.691	-0.043
qNTM2_A07	7	1577.01	20,409,573	20,908,643	16	7.294	0.279	4.213	-3.632
qDFF 1_A07	7	1675.02	20,409,573	20,908,643	16	5.485	0.265	1.141	-2.796
qSI1_A07	7	1741.02	20,409,573	20,908,643	16	2.243	0.329	-0.944	0.964
qPH1_A09	9	316.01	7,600,106	7,941,626	13	6.202	0.211	7.322	14.342
qMP1_A09	9	389.01	7,600,106	9,325,666	53	3.109	0.355	0.590	-0.479
qWC2_A09	9	21.01	1,797,372	1,797,391	0	4.175	0.139	-13.131	29.548
qNTM3_A13	13	403.01	27,648,445	27,648,458	0	6.999	0.279	4.213	-3.613
qLA1_A13	13	405.01	25,062,362	36,817,044	93	6.080	0.070	-3.979	-0.776
qGOT1_A13	13	453.01	30,387,450	32,522,284	36	2.854	0.090	-0.786	-1.760
qSL1_D04	16	176.01	17,437,498	17,437,519	0	3.660	0.087	-0.520	-0.858
qPH2_D06	18	1333.01	51,440,011	52,190,178	25	4.860	0.146	7.251	12.881
qNTM4_D06	18	883.01	20,169,089	31,782,006	286	7.073	0.279	4.213	-3.634
qSL2_D06	18	1054.01	25,802,573	27,002,904	42	5.010	0.198	-0.846	-0.447
qSI2_D06	18	1101.01	17,853,772	28,059,537	301	5.350	0.341	-0.952	0.122
qNTM5_D08	20	170.01	4,036,376	23,854,991	636	11.560	0.263	-4.090	5.870
qLA2_D08	20	165.01	14,189,758	16,109,080	31	4.694	0.108	-5.654	7.207
qLI1_D08	20	325.01	28,201,413	28,201,423	0	3.055	0.176	0.233	0.004
qFP1_D09	21	79.01	662,187	5,281,519	166	5.944	0.298	-2.204	-0.684

### Candidate genes

A total of 211 and 166 genes were identified for whitefly resistance and flowers per plant, respectively, and only a subset was directly associated with these traits, leading to the use of BlastX searches with TAIR to identify genes homologous to known whitefly resistance genes in *Arabidopsis thaliana* within the identified gene set^119^. The mRNA sequence of the Mi 1.2 gene (NM_001247134.1) was depicted via BLASTX search against the TAIR 10 protein database to identify homologous genes in *A. thaliana*, followed by a similar BLASTX search of 211 *G. hirsutum* genes to identify homologs of known whitefly resistance genes, revealing 10 potential candidate genes for whitefly resistance Supplementary Table 1. Additionally, 10 of the 166 genes were directly associated with flowers per plant^120^ (Supplementary Table 2).

## Discussion

Our comprehensive QTL mapping study offers significant insights into the genetic architecture of whitefly resistance and agronomic traits in Gossypium hirsutum^121^. By conducting an extensive composite interval mapping analysis on an F₂ segregating population, we identified multiple stable QTLs that exhibit significant phenotypic effects^122^. This includes five loci responsible for NTM and two primary QTLs (qWC1_A04 and qFP1_D09) linked to WC and FP respectively. The statistical robustness of these findings is substantiated by rigorous permutation testing (1000 iterations), with qNTM_D08 standing out as particularly significant due to its high LOD score (8.67) and substantial contribution to phenotypic variance (27.93%). These results significantly advance our understanding of cotton genetics, with the chromosomal distribution patterns of identified QTLs both confirming known gene clusters for defense responses and revealing novel genetic associations^123^. Of particular interest is the qWC1_A04 locus on chromosome A04, which contains homologs of the well-characterized Mi-1.2 resistance gene from tomato, suggesting evolutionary conservation of defense mechanisms against sap-sucking insects across divergent plant species^124^.

The biological interpretation of our candidate gene analysis reveals a sophisticated, multi-tiered defense system in cotton. The presence of LRR domain-containing proteins (At4g27190 and RPPL1) within QTL intervals strongly suggests the operation of pathogen-associated molecular pattern (PAMP)-triggered immunity^125^, while XA21-like genes likely participate in intracellular kinase signaling cascades that amplify defense responses^126^. Furthermore, the co-localization of secondary metabolism genes with resistance QTLs indicates probable biosynthesis of antixenosis compounds such as terpenoids and flavonoids, which may deter whitefly feeding and oviposition^127^. For floral development traits, the identification of GUS1 within qFP1_D09 provides compelling evidence for hormonal regulation of flowering, as this gene’s known functions in auxin and cytokinin metabolism directly influence floral meristem initiation and differentiation^128^. Similarly, MBDL4’s critical role in managing oxidative stress likely protects developing reproductive tissues from pest-induced physiological damage, thereby maintaining yield potential under infestation pressure^129^.

All these findings about whitefly resistance genes are in line with previous results including, WRKY 40 and Copper protein genes plays role as hub genes against whitefly in cotton^130^ secondary metabolites^131^ defense protein like *CYS6* in tobacco^132^, *β-glucosidase* in squash plants^133^, upregulation of *β-1*,*3-glucanase*,* chitinase* and *peroxidase* in tomato^134^, up regulation of polyphenol oxidase in cucumber^135^ and peroxidase and polyphenol oxidase in tomato and soybean plants^136^. While these findings represent significant advances, several methodological considerations warrant discussion. The F₂ population design, while valuable for initial QTL detection, presents certain limitations regarding effect size estimation accuracy due to residual heterozygosity^137^. Additionally, our single-environment study design precludes assessment of QTL stability across different growing conditions, an important consideration for breeding applications^138^. The relatively large physical intervals of some QTLs, particularly the 19.1 Mb span of qWC1_A04, necessitate future fine-mapping efforts to distinguish true causal variants from linked neutral polymorphisms^139^. These limitations notwithstanding, our results provide a strong foundation for subsequent research and practical applications.

Looking forward, several promising research directions emerge from this work. Development of recombinant inbred line populations will enable more precise QTL validation and effect size estimation, while CRISPR-Cas9 genome editing of prioritized candidate genes (particularly At4g27190 and RPPL1) can establish definitive proof of function^35^. Comprehensive field trials across multiple environments and growing seasons will be essential to evaluate QTL stability and genotype-by-environment interactions^140^. Complementary transcriptomic analysis comparing resistant and susceptible lines under infestation conditions will further elucidate the gene regulatory networks underlying these traits^141^. From a practical breeding perspective, these findings immediately enable marker-assisted selection using the robustly identified qWC1_A04 and qFP1_D09 loci, while also informing strategic pyramiding of complementary resistance mechanisms^142^. The well-supported candidate genes identified here also provide excellent targets for precision breeding approaches, including both transgenic and cisgenic strategies^143^. The integration of these genomic tools with conventional breeding methodologies promises to accelerate the development of whitefly-resistant cotton cultivars with improved yield stability and reduced pesticide dependence, addressing a critical need in sustainable cotton production systems worldwide^144^.

## Conclusion

Our study reveals the complex genetic architecture underlying whitefly resistance and yield in cotton. By utilizing GBS, we identified significant QTLs and candidate genes associated with these traits. Genes such as At4g27190 and RPPL1, which are involved in biological processes such as ADP binding, protein binding, LRR-mediated signal transduction, cell adhesion, DNA repair, transcription, and immune responses in Arabidopsis, suggest their potential importance in cotton defense against sucking pests such as whiteflies. The genes associated with flower development, GUS1, and stress tolerance, MBD4L, contribute to increased flower production and overall plant vigor. The identification of these genes provides valuable insights for developing whitefly resistant cotton cultivars with improved yield potential. Marker-assisted selection and gene editing techniques can be employed to introduce these beneficial traits into elite cotton cultivars. To confirm the precise role of candidate genes in whitefly resistance and yield-related traits, further functional validation, such as gene expression analysis and overexpression and knockout experiments, is necessary. A deeper understanding of their molecular mechanisms, including gene and protein interactions, is essential. Combining whitefly resistance with other desirable traits, such as drought tolerance, heat stress tolerance and fiber quality, can lead to the development of multitrait-resistant cotton varieties. The research presented here provides a strong foundation for developing sustainable and high-yielding cotton cultivars that can effectively combat whitefly infestations and adverse environmental conditions.

## Supplementary Information

Below is the link to the electronic supplementary material.

Supplementary Material 1

## Data Availability

Sequence data that support the findings of this study have been deposited in BankIt (NCBI) with the following accession: Numbers: BankIt2913398 Gh_A04G053800 PQ878369, BankIt2913398 Gh_A04G054700 PQ878370, BankIt2913398 Gh_D09G003400 PQ878371, BankIt2913398 Gh_D09G007300 PQ878372.

## References

[1] Pocrnic, I., Lourenco, D. & Misztal, I. Single nucleotide polymorphism profile for quantitative trait nucleotide in populations with small effective size and its impact on mapping and genomic predictions. *Genetics* **227** (4), iyae103 (2024). 38913695 10.1093/genetics/iyae103 PMC11304960

[2] Sharma, S. K. et al. Genotyping-by-sequencing targets genic regions and improves resolution of genome-wide association studies in autotetraploid potato. *Theor. Appl. Genet.* **137** (8), 180 (2024). 38980417 10.1007/s00122-024-04651-8 PMC11233353

[3] Deka, P. C. Gene cloning. In *Research Anthology on Bioinformatics, Genomics, and Computational Biology*. 650–689 (IGI Global, 2023).

[4] Farzad, N. et al. Spatially resolved epigenome sequencing via Tn5 transposition and deterministic DNA barcoding in tissue. *Nat. Protoc.***19**, 3389–3425 (2024).38943021 10.1038/s41596-024-01013-y

[5] Hu, Y. et al. Plasmodium Vivax populations in the Western greater Mekong subregion evaluated using a genetic barcode. *PLoS Negl. Trop. Dis.* **18** (7), e0012299 (2024). 38959285 10.1371/journal.pntd.0012299 PMC11251639

[6] Satam, H. et al. Next-Generation sequencing technology: current trends and advancements. *Biology (Basel)*. **12** (7), 997 (2023). 37508427 10.3390/biology12070997 PMC10376292

[7] Chakrawarti, N., Deo, I. & Verma, R. Chapter 20 Next-generation sequencing-revolutionizing genomic research. In De Gruyter (eds) *Sustainable Agriculture*. 379–396. (2024).

[8] Nyirahabimana, F. & Solmaz, İ. Cutting-edge of genotyping by sequencing (GBS) for improving drought and salinity stress tolerance in watermelon (Citrullus Lanatus L.): A review. *Plant Mol. Biol Rep.* (2024).

[9] Quail, M. A., Corton, C., Uphill, J., Keane, J. & Gu, Y. Identifying the best PCR enzyme for library amplification in NGS. *Microb. Genomics*. **10** (4), 001228 (2024). 10.1099/mgen.0.001228 PMC11092157 38578268

[10] Rasheed, A., Xia, X. & He, Z. Evolution in the genotyping platforms for plant breeding. 65–78. (2024).

[11] Getachew, S. E., Bille, N. H., Bell, J. M. & Gebreselassie, W. Genotyping by sequencing for plant Breeding- A review. *Adv. Biotechnol. Microbiol.* **14** (4), 555891 (2019).

[12] Nguyen, T. K., Yu, J., Choi, H. W., In, B. C. & Lim, J. H. Optimization of Genotyping-by-sequencing (GBS) in chrysanthemums: selecting proper restriction enzymes for GBS library construction. *Hortic. Sci. Technol.* **36** (1), 108–114 (2018).

[13] Galià-Camps, C., Pegueroles, C., Turon, X., Carreras, C. & Pascual, M. Genome composition and GC content influence loci distribution in reduced representation genomic studies. *BMC Genom.* **25** (1), 410 (2024). 10.1186/s12864-024-10312-3 PMC11046876 38664648

[14] Zhang, S. et al. Genotyping-by-Sequencing of Gossypium hirsutum races and cultivars uncovers novel patterns of genetic relationships and domestication footprints. *Evol. Bioinforma*. **15**, 1176934319889948 (2019). 10.1177/1176934319889948 PMC6868568 31798299

[15] Nazzicari, N., Franguelli, N., Ferrari, B., Pecetti, L. & Annicchiarico, P. The effect of genome parametrization and SNP marker subsetting on genomic selection in autotetraploid alfalfa. *Genes (Basel)*. **15** (4), 449 (2024). 38674384 10.3390/genes15040449 PMC11050091

[16] Hoffmann, A., Timm, A., Johnson, C., Rupp, S. & Grumaz, C. Automation of customizable library Preparation for next-generation sequencing into an open microfluidic platform. *Sci. Rep.* **14** (1), 17150 (2024). 39060329 10.1038/s41598-024-67950-6 PMC11282295

[17] Micheel, J., Safrastyan, A., Aron, F. & Wollny, D. Exploring the impact of primer length on efficient gene detection via high-throughput sequencing. *Nat. Commun.* **15** (1), 5858 (2024). 38997264 10.1038/s41467-024-49685-0 PMC11245535

[18] Nanda, S. R. & Bhattacharyya, S. Genetics and plant breeding vistas-emerging trends in genetics and plant breeding. (2024).

[19] Yadav, S., Yadav, N., Khurana, S. M. P. & Yadav, D. K. Barcoding of life for detection and diagnosis of diseases and pests in potato. In *Approaches for Potato Crop Improvement and Stress Management*. 445–477. (Springer, 2024).

[20] Zhang, H. et al. Optimization of Genotyping-by‐Sequencing (GBS) for germplasm fingerprinting and trait mapping in Faba bean. *Legum Sci.* **6** (3), e254 (2024).

[21] Al-Shuhaib, M. B. S. Classification of Single-Nucleotide Polymorphisms (SNPs): Tips from the Basic Knowledge to the Clinical Outcomes. (2024).

[22] Johnson, M. S., Venkataram, S. & Kryazhimskiy, S. Best practices in Designing, Sequencing, and identifying random DNA barcodes. *J. Mol. Evol.* **91** (3), 263–280 (2023). 36651964 10.1007/s00239-022-10083-z PMC10276077

[23] Pootakham, W. Genotyping by sequencing (GBS) for genome-wide SNP identification in plants. 1–8. (2023). 10.1007/978-1-0716-3024-2_1 36781631

[24] Hussain, S. B., Yousaf, T. & Zubair, M. Determination of genetic divergence pattern of white fly resistant cotton cultivars by using microsatellite. *Asian J. Biochem. Genet. Mol. Biol.* 1–6. (2022).

[25] Samir, S. Human DNA mutations and their impact on genetic disorders. *Recent. Pat. Biotechnol.* **18** (4), 288–315 (2024). 37936448 10.2174/0118722083255081231020055309

[26] Yasir, M. et al. Status and prospects of genome-wide association studies in cotton. *Front. Plant. Sci.* **13**, 1019347 (2022). 36330239 10.3389/fpls.2022.1019347 PMC9623101

[27] Ahmed, H. G. M., Zeng, Y., Yang, X., Fatima, N. & Faisal, A. Crop genome sequencing and their application for crop improvement. In *OMICs-based Techniques for Global Food Security*. 1–26 (Wiley, 2024).

[28] Group, S. P., Reserved, A. R., Group, S. P. & Columbia, B. Cotton genomics and genetics. Vol.15 http://cropscipublisher.com/index.php/cgg (2024).

[29] Sun, L. et al. Construction of host plant insect-Resistance mutant library by High‐Throughput CRISPR/Cas9 system and identification of A Broad‐Spectrum insect resistance gene. *Adv. Sci.* **11** (4), 2306157 (2024). 10.1002/advs.202306157 PMC10811493 38032126

[30] Li, D. et al. Plant resistance against whitefly and its engineering. *Front. Plant. Sci.* **14**, 1232735 (2023). 37711302 10.3389/fpls.2023.1232735 PMC10498545

[31] Dwivedi, N., Mishra, M., Sharma, S. S. & Singh, R. K. Genetic analysis and QTLs identification for resistance to the begomovirus causing pepper leaf curl virus (PepLCV) disease. *J. Plant. Biochem. Biotechnol.* (2023).

[32] Nadeem, S. et al. A comprehensive review on Gossypium hirsutum resistance against cotton leaf curl virus. *Front. Genet.* **15**, 1306469 (2024). 38440193 10.3389/fgene.2024.1306469 PMC10909863

[33] Singh, D. et al. Genetic mapping of Stripe rust resistance in a geographically diverse barley collection and selected biparental populations. *Front. Plant. Sci.* **15**, 1352402 (2024). 39104841 10.3389/fpls.2024.1352402 PMC11299494

[34] Kushanov, F. N. et al. Genetic Diversity, QTL Mapping, and Marker-Assisted selection technology in cotton (Gossypium spp). *Front. Plant. Sci.* 12. (2021). 10.3389/fpls.2021.779386 PMC8716771 34975965

[35] Raj, S. R. G. & Nadarajah, K. QTL and candidate genes: techniques and advancement in abiotic stress resistance breeding of major cereals. *Int. J. Mol. Sci.* **24** (1), 6 (2022). 36613450 10.3390/ijms24010006 PMC9820233

[36] Naqvi, R. Z., Mahmood, M. A., Mansoor, S., Amin, I. & Asif, M. Omics-driven exploration and mining of key functional genes for the improvement of food and fiber crops. *Front. Plant. Sci.* **14**, 1273859 (2024). 38259913 10.3389/fpls.2023.1273859 PMC10800452

[37] Atim, J. et al. Identifying cassava genotypes resistant to the African cassava Whitefly, bemisia tabaci (Gennadius). *Agriculture* **14** (7), 1016 (2024).

[38] Hoque, A., Anderson, J. V. & Rahman, M. Genomic prediction for agronomic traits in a diverse flax (Linum usitatissimum L.) germplasm collection. *Sci. Rep.* **14** (1), 3196 (2024). 38326469 10.1038/s41598-024-53462-w PMC10850546

[39] Kohli, M. et al. Genome-wide association studies for earliness, MYMIV resistance, and other associated traits in Mungbean (Vigna radiata L. Wilczek) using genotyping by sequencing approach. *PeerJ* **12**, e16653 (2024). 38288464 10.7717/peerj.16653 PMC10823994

[40] Bihani, D., Khuman, A. & Chaudhary, B. Comparative transcriptomics reveals novel Spatial gene expression profiles in cotton (Gossypium hirsutum L.) under herbivory and drought stress. *J. Plant. Growth Regul.* **43**, 4018–4037 (2024).

[41] Razzaq, A. et al. Cotton germplasm improvement and progress in Pakistan. *J. Cott Res.* **4** (1), 1 (2021).

[42] Rahman, O. U., Hussain, S. B. & Javed, M. Whitefly-resistant population development source selection among cotton germplasms of Pakistan through multivariate tools. *Plant Mol. Biol. Rep.* (2025).

[43] Supritha, D. S. R., Patil, R. S., Kasturi, S. V. H. & Patil, B. R. Genetic dissection of flowering and plant architectural traits to develop early maturing compact upland cotton genotypes for High-Density planting. *Trop. Plant. Biol.* **18** (1), 1 (2025).

[44] Mahmood, T. et al. Genetic potential and inheritance pattern of phenological growth and drought tolerance in cotton (Gossypium hirsutum L). *Front. Plant. Sci.* **12**, 705392 (2021). 34630456 10.3389/fpls.2021.705392 PMC8497812

[45] Bohra, A. et al. Past and future of cytoplasmic male sterility and heterosis breeding in crop plants. *Plant. Cell. Rep.* **44** (2), 33 (2025). 39841239 10.1007/s00299-024-03414-5

[46] Paul, D. et al. Heterosis breeding and hybrid seed production in cotton. In *Hybrid Seed Production for Boosting Crop Yields*. 225–246 (Springer, 2025).

[47] Khan, B. Effect of varying planting density on weed infestation, crop phenology, yield, and fiber quality of cotton under different sowing methods. *Pure Appl. Biol.* **10** (3), 676–691 (2021).

[48] Jayaram, H., Mahadevegowda, L. G. & Boregowda, M. H. Seri-Entrepreneurship: current status and potential opportunities. *Curr. Agric. Res. J.* **12** (1), 385–399 (2024).

[49] Aparicio, J., Kapelko, M. & Zofío, J. L. The measurement of environmental economic inefficiency with pollution-generating technologies. *Resour. Energy Econ.* **62**, 101185 (2020).

[50] Ali, Q. et al. A modified protocol for rapid DNA isolation from cotton (Gossypium spp). *MethodsX* **6**, 259–264 (2019). 30792967 10.1016/j.mex.2019.01.010 PMC6370549

[51] Ishaq, M. Z. et al. Effect of sowing dates and genotypes on yield and yield contributing traits of upland cotton (Gossypium hirsutum L). *Sarhad J. Agric.* **38** (1), 16–25 (2021).

[52] Khan, M. R. J., Ullah, K., Khan, M. M. & Khan, R. Impact of earliness, flowering time and plant design on late sown cotton production. (2024).

[53] Getman-Pickering, Z. L. et al. (ed, S.) LeafByte: A mobile application that measures leaf area and herbivory quickly and accurately. *Methods Ecol. Evol.* **11** 2 215–221 (2020).

[54] Shah, S. H. J., Paredes-Montero, J. R., Malik, A. H., Brown, J. K. & Qazi, J. Distribution of bemisia tabaci (Gennadius) (Hemiptera: Aleyrodidae) mitotypes in commercial cotton fields in the Punjab Province of Pakistan. *Fla. Entomol.* **103** (1), 41–47 (2020).

[55] Zafar, M. M. et al. Investigation of salt tolerance in cotton germplasm by analyzing agro-physiological traits and ERF genes expression. *Sci. Rep.* **14** (1), 11809 (2024). 38782928 10.1038/s41598-024-60778-0 PMC11116465

[56] Raza, I. et al. Correlation analysis of stem hardness traits with fiber and yield-related traits in core collections of Gossypium hirsutum. *J. Cott Res.* **4** (1), 8 (2021).

[57] Jansson, L. et al. Assessment of DNA quality for whole genome library Preparation. *Anal. Biochem.* **695**, 115636 (2024). 39111682 10.1016/j.ab.2024.115636

[58] Babafemi, A. I., Temitope, O., Popoola, J. O. & Conrad, O. A. Comparison of two extraction methods to obtain quality genomic DNA from eggplants (Solanum sp.). In *Biotechnological Approaches To Sustainable Development Goals* 305–315. (Springer, 2023).

[59] Wilkinson, R. C. et al. Determining the efficacy of disinfectants at nucleic acid degradation. *J. Appl. Microbiol.* **134** (11), lxad244 (2023). 37884448 10.1093/jambio/lxad244

[60] Kumar, K. R., Cowley, M. J. & Davis, R. L. *Next-Generation Sequencing and Emerging Technologies** (Semin Thromb Hemost, 2024). 10.1055/s-0044-1786397 38692283

[61] LaBonte, N. R. et al. Improving precision and accuracy of genetic mapping with genotyping‐by‐sequencing data in outcrossing species. *GCB Bioenergy* **16** (7), e13167. (2024).

[62] Pradhan, S. P., Rout, A. K., Rao, E. V. & Pradhan, S. K. Genomics data analysis techniques in Bioinformatics. In *Current Trends in Fisheries Biotechnology*. 139–152. (Springer, 2024).

[63] Cheng, Y. et al. Gossypium purpurascens genome provides insight into the origin and domestication of upland cotton. *J. Adv. Res.* **56**, 15–29 (2024). 36966917 10.1016/j.jare.2023.03.006 PMC10834806

[64] Torkamaneh, D., Laroche, J. & Belzile, F. Genome-wide SNP calling from genotyping by sequencing (GBS) data: A comparison of seven pipelines and two sequencing technologies. *PLoS One*. **11** (8), 1–14 (2016). 10.1371/journal.pone.0161333 PMC4993469 27547936

[65] Vasilita, C. et al. Express barcoding with NextGenPCR and minion for species-level sorting of ecological samples. *Mol. Ecol. Resour.* **24** (3), 1–7 (2024). 10.1111/1755-0998.13922 38240168

[66] Shui, G. et al. Identification of SSR markers linked to the abscission of cotton boll traits and mining germplasm in cotton. *J. Cott Res.* **7** (1), 20 (2024).

[67] Buabeng, I. Bucknell digital commons genetic diversity of paxistima canbyi A. Gray: conserving A rare plant species endemic to the Eastern united States. (2024).

[68] Ma, J. et al. Deciphering the dynamic expression network of fiber elongation and the functional role of the GhTUB5 gene for fiber length in cotton based on an introgression population of upland cotton. *J. Adv. Res.* (2024) 10.1016/j.jare.2024.08.004 PMC12225886 39106927

[69] Brhane, H. & Hammenhag, C. Genetic diversity and population structure analysis of a diverse panel of pea (Pisum sativum). *Front. Genet.* **15**, 1–21 (2024). 10.3389/fgene.2024.1396888 PMC11169732 38873115

[70] Damien, S., ASGNDLF, E. & Votava 2 and Hussein A., H. *Genetic Diversity and Population Structure of a Large USDA Sesame Collection*. (2024). 10.3390/plants13131765 PMC11243581 38999604

[71] Ma, Z. et al. High-quality genome assembly and resequencing of modern cotton cultivars provide resources for crop improvement. *Nat. Genet.* **53** (9), 1385–1391 (2021). 34373642 10.1038/s41588-021-00910-2 PMC8423627

[72] Pandey, A. et al. Multi-GWAS reveals significant genomic regions for Mungbean yellow mosaic India virus resistance in Urdbean (Vigna mungo (L.) across multiple environments. *Plant. Cell. Rep.* **43** (7), 166 (2024). 38862789 10.1007/s00299-024-03257-0

[73] Zayas, G. A., Rodriguez, E., Hernandez, A., Rezende, F. M. & Mateescu, R. G. Breed of origin analysis in genome-wide association studies: enhancing SNP-based insights into production traits in a commercial Brangus population. *BMC Genom.* **25** (1), 654 (2024). 10.1186/s12864-024-10465-1 PMC11218112 38956457

[74] Zhang, S. et al. Genotyping-by-Sequencing of Gossypium hirsutum races and cultivars uncovers novel patterns of genetic relationships and domestication footprints. *Evol. Bioinforma*. **15**, 117693431988994 (2019). 10.1177/1176934319889948 PMC6868568 31798299

[75] Le Meur, A. et al. Tools for short variant calling and the way to deal with big datasets. In *Phylogenomics* 219–250. (Elsevier, 2024).

[76] Wragg, D. et al. A cautionary Tale of low-pass sequencing and imputation with respect to haplotype accuracy. *Genet. Sel. Evol.* **56** (1), 6 (2024). 38216889 10.1186/s12711-024-00875-w PMC10785484

[77] Abbas, M. et al. Insights into genetic diversity and functional significance of the bHLH genes in cotton fiber development. *Ind. Crops Prod.* **216**, 118763 (2024).

[78] Felício, D. et al. Integrating functional scoring and regulatory data to predict the effect of non-coding SNPs in a complex neurological disease. *Brief. Funct. Genomics*. **23** (2), 138–149 (2024). 37254524 10.1093/bfgp/elad020

[79] Feng, C., Stetina, S. R. & Erpelding, J. E. Transcriptome analysis of resistant cotton germplasm responding to reniform nematodes. *Plants* **13** (7), 958 (2024). 38611488 10.3390/plants13070958 PMC11013486

[80] Chen, Z., Ain, N., ul, Zhao, Q. & Zhang, X. From tradition to innovation: conventional and deep learning frameworks in genome annotation. *Brief. Bioinform*. **25** (3), bbae138 (2024). 38581418 10.1093/bib/bbae138 PMC10998533

[81] Niewold, T. B., Aksentijevich, I., Gorevic, P. D., Gibson, G. & Yao, Q. Genetically transitional disease: conceptual Understanding and applicability to rheumatic disease. *Nat. Rev. Rheumatol.* **20** (5), 301–310 (2024). 38418715 10.1038/s41584-024-01086-9 PMC13175155

[82] Schwertfirm, G. et al. Genome-wide association study revealed significant SNPs for anthracnose resistance, seed alkaloids and protein content in white lupin. *Theor. Appl. Genet.* **137** (7), 155 (2024). 38858311 10.1007/s00122-024-04665-2 PMC11164739

[83] Wu, K. et al. Exploring noncoding variants in genetic diseases: from detection to functional insights. *J. Genet. Genomics*. **51** (2), 111–132 (2024). 38181897 10.1016/j.jgg.2024.01.001

[84] Casas, L. & Saborido-Rey, F. A review of genomics methods and bioinformatics tools for the analysis of close-kin mark-recapture. *Front. Mar. Sci.* **10**, 1–15 (2023).

[85] Rachappanavar, V., Padiyal, A., Sharma, J. K. & Negi, N. Analytical pipelines for the GBS analysis. In *Genotyping by Sequencing for Crop Improvement*. 161–187 (Wiley, 2022).

[86] Ruidong, S. et al. Identification of QTLs and their candidate genes for the number of maize Tassel branches in F2 from two higher generation sister lines using QTL mapping and RNA-seq analysis. *Front. Plant. Sci.* **14**, 1202755 (2023). 37641589 10.3389/fpls.2023.1202755 PMC10460468

[87] Kaushal, S. et al. Enhancing the potential of phenomic and genomic prediction in winter wheat breeding using high-throughput phenotyping and deep learning. *Front. Plant. Sci.* **15**, 1410249 (2024). 38872880 10.3389/fpls.2024.1410249 PMC11169824

[88] Perea, C. et al. Bioinformatic analysis of genotype by sequencing (GBS) data with NGSEP. *BMC Genom.* **17** (S5), 498 (2016). 10.1186/s12864-016-2827-7 PMC5009557 27585926

[89] Torkamaneh, D., Laroche, J., Bastien, M., Abed, A. & Belzile, F. Fast-GBS: a new pipeline for the efficient and highly accurate calling of SNPs from genotyping-by-sequencing data. *BMC Bioinform.* **18** (1), 5 (2017). 10.1186/s12859-016-1431-9 PMC5210301 28049422

[90] Taniguti, C. H. et al. Developing best practices for genotyping-by-sequencing analysis in the construction of linkage maps. *Gigascience* **12**, giad092 (2022). 37889010 10.1093/gigascience/giad092 PMC10603770

[91] Torkamaneh, D., Laroche, J., Belzile, F. & Genome-Wide, S. N. P. Calling from genotyping by sequencing (GBS) data: A comparison of seven pipelines and two sequencing technologies. *PLoS One* **11** (8), e0161333. (2016). 10.1371/journal.pone.0161333 PMC4993469 27547936

[92] Hicks, J., Stuber, T., Lantz, K., Torchetti, M. & Robbe-Austerman, S. vSNP: a SNP pipeline for the generation of transparent SNP matrices and phylogenetic trees from whole genome sequencing data sets. *BMC Genom.* **25** (1), 545 (2024). 10.1186/s12864-024-10437-5 PMC11143592 38822271

[93] Elshire, R. J. et al. A robust, simple genotyping-by-sequencing (GBS) approach for high diversity species. *PLoS One* **6** (5), e19379 (2011). 10.1371/journal.pone.0019379 PMC3087801 21573248

[94] Martínez-Guardiola, C., Parreño, R. & Candela, H. MAPtools: command-line tools for mapping-by-sequencing and QTL-Seq analysis and visualization. *Plant. Methods*. **20** (1), 107 (2024). 39014443 10.1186/s13007-024-01222-2 PMC11253474

[95] Qin, M. F. et al. Construction of a high-density bin-map and identification of fruit quality-related quantitative trait loci and functional genes in Pear. *Hortic. Res.* **9**, uhac141 (2022). 36072841 10.1093/hr/uhac141 PMC9437719

[96] Tan, H. Z. et al. A high-density linkage map reveals broad- and fine-scale sex differences in recombination in the hihi (stitchbird; Notiomystis cincta). Heredity (Edinb). (2024). 10.1038/s41437-024-00711-3 PMC11437212 39095652

[97] Majhi, P. K. et al. Genetic mapping of valued genes with significant traits in crop plants. In *Bioinformatics for Plant Research and Crop Breeding*. 99–134 (Wiley, 2024).

[98] Naik, S. et al. Genome-wide SNP discovery and genotyping delineates potential QTLs underlying major yield‐attributing traits in buckwheat. *Plant. Genome* **17** (1), e20427. (2024). 10.1002/tpg2.20427 PMC12807168 38239091

[99] Wang, X. et al. Effect of genotyping errors on linkage map construction based on repeated chip analysis of two Recombinant inbred line populations in wheat (Triticum aestivum L). *BMC Plant. Biol.* **24** (1), 306 (2024). 38644480 10.1186/s12870-024-05005-8 PMC11034145

[100] Kaur, H., Kumar, V., Pathak, D. & Sangha, M. K. Antibiosis mechanism and bases of resistance to the whitefly, bemisia tabaci (Gennadius) in upland cotton introgression lines. *Phytoparasitica* **52** (2), 42 (2024).

[101] Shaikh, T. et al. QTL mapping to identify loci and candidate genes associated with freezing tolerance trait in camelina sativa. *Ind. Crops Prod.* **222**, 119562 (2024).

[102] Boopathi, N. M. et al. Identification of stable and multiple environment interaction QTLs and candidate genes for fiber productive traits under irrigated and water stress conditions using intraspecific RILs of Gossypium hirsutum var. MCU5 X TCH1218. *Front. Plant. Sci.* **13**, 851504 (2022). 35519814 10.3389/fpls.2022.851504 PMC9062235

[103] Magwanga, R. O. et al. Identification of QTLs and candidate genes for physiological traits associated with drought tolerance in cotton. *J. Cott Res.* **3** (1), 3 (2020).

[104] Mahfouze, S. A. Detection of The Mi-1. 2 Gene from tomato confers resistance against whitefly (Bemisia tabaci). (2017).

[105] Qasim Aslam, M. et al. Cotton Mi-1.2-like gene: A potential source of whitefly resistance. *Gene* **851**, 146983 (2023). 36270457 10.1016/j.gene.2022.146983

[106] de Ubert, P. & Nava, I. Genetic mapping and comparative analysis of heading date in hexaploid oat. *Euphytica* **220** (11), 176 (2024).

[107] Talukder, Z. I. et al. Genetic analysis of basal stalk rot resistance introgressed from wild Helianthus petiolaris into cultivated sunflower (Helianthus annuus L.) using an advanced backcross population. *Front. Plant. Sci.* **14**, 1278048 (2023). 37920712 10.3389/fpls.2023.1278048 PMC10619160

[108] Kumar, N. V. M. et al. 63K SNP chip based linkage mapping and QTL analysis for fibre quality and yield component traits in Gossypium Barbadense L. cotton. *Euphytica* **215** (1), 6 (2019).

[109] Sauvage, C., Vagner, M., Derôme, N., Audet, C. & Bernatchez, L. Coding gene single nucleotide polymorphism mapping and quantitative trait loci detection for physiological reproductive traits in brook Charr, Salvelinus Fontinalis. *G3 Genes|Genomes|Genetics*. **2** (3), 379–392 (2012). 22413092 10.1534/g3.111.001867 PMC3291508

[110] Wang, X. et al. An SNP-Based genetic map and QTL mapping for growth traits in the Red-Spotted grouper (Epinephelus akaara). *Genes (Basel)*. **10** (10), 793 (2019). 31614822 10.3390/genes10100793 PMC6826704

[111] Diouf, L. et al. QTL mapping of fiber quality and Yield-Related traits in an Intra-Specific upland cotton using genotype by sequencing (GBS). *Int. J. Mol. Sci.* **19** (2), 441 (2018). 29389902 10.3390/ijms19020441 PMC5855663

[112] Ablamowicz, R. An abstract of the thesis of. *Young***c(1)**, 105–106 (2007).

[113] Deng, X. et al. QTL mapping for fiber quality and yield-related traits across multiple generations in segregating population of CCRI 70. *J. Cott Res.* **2** (1), 13 (2019).

[114] Keerio, A. et al. QTL mapping for fiber quality and yield traits based on introgression lines derived from Gossypium hirsutum × G. tomentosum. *Int. J. Mol. Sci.* **19** (1), 243 (2018). 29342893 10.3390/ijms19010243 PMC5796191

[115] Rawandoozi, Z. et al. Pedigree-based QTL analysis of flower size traits in two multi-parental diploid Rose populations. *Front. Plant. Sci.* **14**, 1226713 (2023). 37650001 10.3389/fpls.2023.1226713 PMC10464838

[116] Zhang, J. et al. QTL and candidate gene identification of the node of the first fruiting branch (NFFB) by QTL-seq in upland cotton (Gossypium hirsutum L). *BMC Genom.* **22** (1), 882 (2021). 10.1186/s12864-021-08164-2 PMC8650230 34872494

[117] Kushanov, F. N. et al. Genetic analysis of mutagenesis that induces the photoperiod insensitivity of wild cotton Gossypium hirsutum Subsp. Purpurascens. *Plants* **11** (22), 3012 (2022). 36432741 10.3390/plants11223012 PMC9698681

[118] Said, J. I., Knapka, J. A. & Song, M. Z. Z. J. Cotton qtldb: a cotton QTL database for QTL analysis, visualization, and comparison between Gossypium hirsutum and G. hirsutum x G. barbadense populations. *Mol. Genet. Genomics*. **290** (4), 1615–1625 (2015). 25758743 10.1007/s00438-015-1021-y

[119] Dubey, N. K. et al. Comparative transcriptome analysis of Gossypium hirsutum L. In *Response to Sap sucking insects : aphid and whitefly*. 1–20 (2013). 10.1186/1471-2164-14-241 PMC3637549 23577705

[120] Liu, Y. X. et al. Genome-wide identification and expression analysis of Tlps gene family under abiotic stresses in camelina sativa using bioinformatics methods. *Appl. Ecol. Environ. Res.* **22** (1), 249–263 (2024).

[121] Khan, M. et al. Unraveling key genes and pathways involved in verticillium wilt resistance by integrative GWAS and transcriptomic approaches in upland cotton. *Funct. Integr. Genomics*. **25** (1), 39 (2025). 39955705 10.1007/s10142-025-01539-8

[122] Jia, X. et al. QTL mapping and candidate gene analysis for cotton fiber quality and early maturity using F2 and F3 generations. *Plants* **14** (7), 1063 (2025). 40219131 10.3390/plants14071063 PMC11991040

[123] Wang, L. et al. Integrative GWAS and transcriptomics reveal GhAMT2 as a key regulator of cotton resistance to verticillium wilt. *Front. Plant. Sci.* **16**, 1495662 (2025). 40353226 10.3389/fpls.2025.1563466 PMC12062179

[124] Aslam, M. Q. et al. Cotton Mi-1.2-like gene: A potential source of whitefly resistance. *Gene* **851**, 146983 (2023). 36270457 10.1016/j.gene.2022.146983

[125] Cai, M., Yu, H., Sun, E. & Zuo, C. Receptor-like proteins: decision-makers of plant immunity. *Phytopathol. Res.* **6** (1), 58 (2024).

[126] Snoeck, S., Johanndrees, O., Nürnberger, T. & Zipfel, C. Plant pattern recognition receptors: from evolutionary insight to engineering. *Nat. Rev. Genet.* **26** (4), 268–278 (2025). 39528738 10.1038/s41576-024-00793-z

[127] Soujanya, P. L., Karjagi, C. G., Suby, S. B., Yathish, K. R. & Sekhar, J. C. Host plant resistance to insect pests in Maize. In *Plant Resistance to Insects in Major Field Crops*. 141–168. (Springer, 2024).

[128] Zhou, L., Iqbal, A., Yang, M. & Yang, Y. Research progress on gene regulation of plant floral organogenesis. *Genes (Basel)*. **16** (1), 79 (2025). 39858626 10.3390/genes16010079 PMC11765145

[129] Abubakar, M., Koul, B., Chandrashekar, K., Raut, A. & Yadav, D. Whitefly (Bemisia tabaci) management (WFM) strategies for sustainable agriculture: A review. *Agriculture* **12** (9), 1317 (2022).

[130] Li, J. et al. Transcriptome analysis reveals a comprehensive insect resistance response mechanism in cotton to infestation by the phloem feeding insect bemisia tabaci (whitefly). *Plant. Biotechnol. J.* **14** (10), 1956–1975 (2016). 26923339 10.1111/pbi.12554 PMC5042180

[131] Luo, M. & Li, B. Non-volatile metabolites mediate plant interactions with insect herbivores. *Plant. J.* **114**, 1164–1177 (2023). 36891808 10.1111/tpj.16180

[132] Du, H. et al. Armet from whitefly saliva acts as an effector to suppress plant defences by targeting tobacco Cystatin. *New. Phytol*. **234** (5), 1848–1862 (2022). 35238409 10.1111/nph.18063

[133] Prasad, K. V. H. Community functions/dynamics. In *Insect Ecology: Concepts to Management*. 163–188. (Springer, 2022).

[134] Zribi, I., Ghorbel, M. & Brini, F. Pathogenesis related proteins (PRs): from cellular mechanisms to plant defense. *Curr. Protein Pept. Sci.* **22** (5), 396–412 (2021). 33390143 10.2174/1389203721999201231212736

[135] Mohamed, H. I. et al. Comparative effectiveness of potential elicitors of soybean plant resistance against spodoptera littoralis and their effects on secondary metabolites and antioxidant defense system. *Gesunde Pflanz*. **73** (3), 273–285 (2021).

[136] Hegazy, F., Eissa, G., Khattab, M., Mesbah, I. & El-Sheikh, M. Alternative control method for the Whitefly, bemisia tabaci (Genn.) (Homoptera: Aleyrodidae) on soybean plants. *J. Plant. Prot. Pathol.* **14**, 267–273 (2023).

[137] Vieira, R. A., Nogueira, A. P. O. & Fritsche-Neto, R. Optimizing the selection of quantitative traits in plant breeding using simulation. *Front. Plant. Sci.* **16**. (2025). 10.3389/fpls.2025.1495662 PMC11847808 39996117

[138] Li, T. et al. Genome-wide association study identifies elite alleles of FLA2 and FLA9 controlling flag leaf angle in rice. *BMC Genom.* **26** (1), 280 (2025). 10.1186/s12864-025-11487-z PMC11927237 40119348

[139] Tura, H. et al. QTL analysis and fine mapping of a QTL for yield-related traits in wheat grown in dry and hot environments. *Theor. Appl. Genet.* **133** (1), 239–257 (2020). 31586227 10.1007/s00122-019-03454-6 PMC7990757

[140] Umani, K. et al. Evaluation of genotype x environment interaction using yield and UAV-based vegetation index data from multi-environment trials in Chickpea. *J. Crop Improv.* :1–26. (2025).

[141] Zhang, X., Wang, X. & Wang, T. Comprehensive transcriptomic analysis reveals Defense-Related genes and pathways of rice plants in response to fall armyworm (Spodoptera frugiperda) infestation. *Plants* **13** (20), 2879 (2024). 39458827 10.3390/plants13202879 PMC11510987

[142] Mores, A. et al. Genomic approaches to identify molecular bases of crop resistance to diseases and to develop future breeding strategies. *Int. J. Mol. Sci.* **22** (11), 5423 (2021). 34063853 10.3390/ijms22115423 PMC8196592

[143] Uno, Y., Yagi, M., Hirakawa, H. & Hosokawa, M. Genetic resources and breeding technology in African violet (Saintpaulia ionantha H. Wendl.). 299–327. (2025).

[144] Nalla, M. K., Schafleitner, R., Pappu, H. R. & Barchenger, D. W. Current status, breeding strategies and future prospects for managing Chilli leaf curl virus disease and associated begomoviruses in Chilli (Capsicum spp). *Front. Plant. Sci.* **14**, 1223982 (2023). 37936944 10.3389/fpls.2023.1223982 PMC10626458

